# Changes in Recombinant Human Bone Morphogenetic Protein-2 Use in Posterior Fusion Over the Past Two Decades

**DOI:** 10.7759/cureus.18055

**Published:** 2021-09-17

**Authors:** Naveed Nabizadeh, Steven D Glassman, Mladen Djurasovic, Charles H Crawford, Jeffrey L Gum, Leah Carreon

**Affiliations:** 1 Orthopaedics, Norton Leatherman Spine Center, Norton Healthcare, Louisville, USA; 2 Orthopaedic Surgery, University of Louisville, Louisville, USA; 3 Research, Norton Leatherman Spine Center, Norton Healthcare, Louisville, USA

**Keywords:** bone morphogenetic protein, posterolateral fusion, transforaminal lumbar intebrody fusion, spine fusion, lumbar fusion

## Abstract

Background

In 2011, studies suggested that complications and cancer rates associated with bone morphogenetic protein (BMP) were greater than previously reported. However, later studies reported complication rates similar to prior literature and no increased cancer rate. We evaluated the pattern of clinical utilization of BMP in posteriorly based lumbar fusion by comparing two periods: 2002-2004 and 2017-2019.

Methods

Patients who received BMP from 2002-2004 (Early) and 2017-2019 (Late) from a single multi-surgeon institution who had a lumbar fusion were identified. One hundred patients from each cohort were randomly selected. Mean total BMP used at each level and the proportion of BMP placed in the interbody space versus posterolateral gutters were evaluated.

Results

In the transforaminal lumbar intebody fusion (TLIF) cohort, the total BMP dose in the Late group (6.15 mg) was nearly half of that used in the Early group (12.04 mg, p<0.000). The amount of BMP used in the posterolateral gutters remained similar (Early: 4.01 mg vs Late: 3.38 mg, p=0.222). The amount of BMP used in the interbody space was less in the Late group (2.76 mg) compared to the Early group (8.03 mg, p<0.000). In the posterior spinal fusion (PSF) cohort, the total BMP dose remained similar between the Early (11.96 mg) and the Late groups (10.82 mg, p=0.007).

Conclusion

Change in the use of BMP in TLIF cases was driven by the complications reported in the literature with no change in outcome. A similar impetus was not seen for PSF.

## Introduction

Bone morphogenetic protein (BMP) was discovered by Marshall Urist in 1965 [1], and after 30 years of basic science study, its effectiveness in spinal fusion was assessed by prospective clinical trials in the late 1990s [2]. BMP was approved by the Food and Drug Administration (FDA) in 2002 for single-level anterior interbody fusion in conjunction with a lumbar tapered fusion cage, to treat degenerative disc disease, Grade I spondylolisthesis, and/or retrolisthesis [2]. Despite these limited “on-label” indications, BMP was primarily used “off-label” in posterolateral and transforaminal interbody fusion applications, accounting for more than 85% of cases [3].

With increased utilization, complications were noted but considered to be in an acceptable range [4,5]. The most commonly reported complications reflected the same proinflammatory and bone induction properties of BMP that led to fusion healing. There were reports of increased wound drainage [6], bony erosion around anterior fusion cages [7], and heterotopic ossification, sometimes causing radiculopathy [8,9]. The most serious concerns were related to anterior cervical fusion complicated by prevertebral edema, airway obstruction, re-intubation, and even death [10]. In 2008, the FDA issued a black box warning to avoid use of recombinant human bone morphogenetic protein-2 (rhBMP-2) in anterior cervical decompression and fusion procedures. Despite these issues, BMP was generally regarded as safe, with perhaps the most significant limitation to BMP use being high cost. Driven in part by that concern, surgeons began to utilize BMP in lower doses. Initial experience with lower BMP doses suggested a decrease in complication rate and cost, without an apparent deterioration in fusion rate [11-13].

The perception of BMP changed dramatically in 2011 with the publication of a special edition of The Spine Journal devoted to the complications of BMP. Several authors suggested that the rate of complications with BMP were dramatically greater than had been previously reported [14]. There was also a study suggesting a high rate of cancer associated with BMP use that had not previously been reported [15]. The assertions in this special edition of The Spine Journal were cited extensively in the media, and the response of both patients and surgeons resulted in a precipitous decrease in BMP utilization.

Over the next several years these issues were investigated extensively through new prospective studies, analysis of large databases, and systematic reviews. The vast majority of the subsequent studies reported complication rates similar to the prior literature and repudiated the assertion that complications had been significantly underreported [16-18]. Importantly, numerous cohort and large database studies demonstrated no association between BMP use and increased cancer rate [19,20].

As the safety concerns regarding BMP application have subsided, widespread utilization has resumed. However, based upon accumulated clinical experience, continued concern with BMP cost [13], and the impact of newer studies, the pattern of BMP use appears to have changed [18]. We attempt to quantify the impact of almost 20 years of clinical experience on the pattern of clinical utilization of BMP in posteriorly based lumbar fusion by comparing two periods of time: 2002-2004 and 2017-2019.

## Materials and methods

After receiving Institutional Review Board Approval, patients who received BMP from years 2002-2004 (Early) and 2017-2019 (Late) from a single multi-surgeon institution were identified. There were 1471 patients in the Early group (2002-2004) and 2809 patients in the Late group (2017-2019). Patients who had cervical, thoracic, and anterior lumbar fusions were excluded. One hundred patients from each cohort were selected using random number generator. Medical charts were reviewed and the following data were collected: demographic data included patient’s age, sex, insurance status, smoking history, surgical levels, the number of fused levels, the amount and site of implanted BMP placement, additional graft material (allograft, demineralized bone matrix), and revision status (primary versus revision). The mean total BMP used at each level and the proportion of BMP placed in the interbody space versus posterolateral gutters were evaluated.

All statistical analyses was performed using IBM SPSS V26.0 (IBM, Armonk, New York) with p value <0.05 being considered statistically significant. Continuous variables were compared using independent t-tests and categorical variables were compared using Fisher's exact test. 

## Results

A total of 400 cases, 100 transforaminal lumbar interbody fusion (TLIF) cases and 100 posterolateral fusion cases in each time period, Early and Late, were included in the study. Demographic and surgical data for the TLIF cases are summarized in Table 1 and show that the cases in the Late group were older (57.3 ± 12.0 years) than the cases in the Early group (49.6 ± 11.4 years, p<0.000). There were also fewer smokers in the Late group (43) compared to the Early group (21, p=0.002).

**Table 1:** Summary of demographic and surgical data for the TLIF cases. BMP, bone morphogenetic protein; TLIF, transforaminal lumbar interbody fusion.

	Early	Late	
	Mean	Mean	p-Value
Age, Years, Mean (SD)	49.6 (11.4)	57.3 (12.0)	0.000
Males, N (%)	41	42	0.775
Smoker	43	21	0.002
Number of Levels			0.173
1	82	84	
2	20	12	
3	0	1	
4	0	2	
Additional Graft			
Iliac Crest Bone Graft	25	2	
Local Bone Graft	85	99	
Allograft	15	22	
MasterGraft	12	0	
Demineralized Bone	34	35	
BMP Dose			
Total	12.04 (1.42)	6.15 (3.52)	<0.000
Interbody, mg	8.03 (3.14)	2.76 (1.85)	<0.000
Posterior, mg	4.01 (2.75)	3.38 (4.29)	0.222
Percent in Interbody	66.6 (22.9)	44.93 (52.56)	0.244
Percent in Posterior	33.72 (22.77)	55.06 (21.58)	0.275
Estimated Blood Loss, mL, Mean (SD)	719.37 (556.55)	324.08 (224.11)	0.000
Operative Time, min, Mean (SD)	228.29 (66.76)	208.71 (73.90)	0.026

The total dose of BMP used in the cases in the Late group (6.15 ± 3.42 mg) was nearly half the total dose used in the cases in the Early group (12.04 ± 1.42 mg, p<0.000). Although the amount of BMP used in the posterolateral gutters remained similar for the cases in the Early (4.01 ±2.75 mg) and Late groups (3.38 ± 4.29 mg, p=0.222), the amount of BMP used in the interbody space was statistically significantly less in the Late group (2.76 ± 1.85 mg) compared to the Early group (8.03 ± 3.14 mg, p<0.000).

Demographic and surgical data for the cases in the posterolateral fusion-only cases are summarized in Table 2. Similar to the TLIF cohorts, the cases in the Late group were older (64.5 ± 10.6 years) than the cases in the Early group (57.8 ± 13.0 years, p<0.000). There were also fewer smokers in the Late group (30) compared to the Early group (18, p=0.068), but this was not statistically significant. Although statistically significantly different (p=0.007), the total BMP dose used in the posterior-only group remained similar between the Early (11.96 ± 2.57 mg) and the Late groups (10.82 ± 3.37 mg).

**Table 2:** Summary of demographic and surgical data for PSF cases. BMP, bone morphogenetic protein; PSF, posterior spinal fusion.

	Early	Late	p-Value
	Mean	Mean	
Age, Years, Mean (SD)	57.88 (12.95)	64.5 (10.62)	0.001
Males, N (%)	41	37	0.664
Smoker	30	18	0.068
Number of Levels			0.025
1	42	41	
2	43	32	
3	12	12	
4	3	15	
Additional Graft			
Iliac Crest Bone Graft	42	12	
Local Bone Graft	83	98	
Allograft	30	35	
MasterGraft	27	12	
Demineralized Bone	26	17	
BMP Dose			
Posterior, mg	11.96 (2.57)	10.82 (3.37)	0.007
Estimated Blood Loss, mL, Mean (SD)	687.75 (439.22)	435.15 (322.10)	0.000
Operative Time, min, Mean (SD)	228.81 (66.89)	210.55 (84.61)	0.096

## Discussion

Following the decline of BMP use in 2011, multiple studies have demonstrated the efficacy and safety of BMP for spinal fusion [13,21,22]. Widespread use has resumed [23,24], but the current study suggests that BMP use for TLIF, in terms of dose and site of application, has changed over the last decade. The total amount of BMP applied per level has decreased to approximately half (12 mg to 6 mg). Aside from this decreased dose, the proportion of BMP used in the interbody space decreased by 22%, from 67% to 45%, with an associated increased proportion used in the posterolateral gutters 33% to 55%. This trend may have been influenced by more recent studies showing the efficiency of lower doses in inducing fusion [13,21,22] and refinement in techniques on the use of BMP in the interbody space. These refinements include placing the BMP-infused sponges away from the dura [23], the use of a sealant to protect the nerve roots prior to insertion of the sponges into the cage [24], and the use of structural allografts as barrier between the disc space and the nerve roots [25,26]. 

In contrast to the trend of using smaller doses in the interbody space, the current study showed that BMP doses used for posterolateral fusion have not changed significantly over the last two decades. The surgeons continue to apply relatively high doses of BMP in posterolateral fusion setting to overcome the risk of non-union due to limited surface for healing, the gap between transverse processes, and the milieu of distractive forces. It remains unclear whether these doses could be reduced without diminution of fusion rate. As the associated cost of BMP is an issue for both TLIF and posterior spine fusion (PSF), this does not seem to be the primary driver for the change in practice pattern. The more substantial alteration in TLIF usage may imply that the concern for complications with TLIF is a greater influence than cost on surgeon behavior.

Aside from the evolving techniques in the use of BMP during surgery, there has also been a refinement in patient selection, with a preferential use of BMP in older patients. The current study shows that over the last decade there has been an increase in the use of BMP in older patients, particularly those over 60 years old. The use of BMP in older patients is advantageous as it has been shown to improve fusion rates [27]. More importantly, the use of BMP obviates the need for bone graft harvest, decreasing operative time and blood loss [27]. This decreased operative time and blood loss has been shown to lower complication rates [28]. The smaller amount of dissection may also lead to a decrease in the need for in-patient rehabilitation [27]. Improvements in surgical techniques are also reflected in the statistically significantly lower blood loss in patients in the Late group for both the PSF and TLIF cohorts.

We also expected an increased use of BMP in smokers as a previous study showed that BMP increases fusion rates in smokers [29,30]. However, the current study showed that there were less smokers in the Late group compared to the Early group in both the TLIF and PSF cohorts. This may be reflective in a change in the general practice, with surgeons encouraging patients to quit smoking prior to any fusion procedures. 

There are limitations in this study. First, this is a retrospective study with a small group from a single institution. Second, alternative BMP carriers for lumbar fusion were not investigated. Patients included in the study were operated on by eight surgeons, who may use different techniques when applying BMP in the interbody space and posterolateral gutters.

## Conclusions

Change in the use of BMP in TLIF cases was driven by the complications reported in the literature. A similar impetus was not seen for PSF. Although spine surgeons continue to use BMP in TLIFs to enhance fusion, BMP is increasingly used for older patients, but with lower doses and smaller amounts placed in the interbody space. Doses used for posterolateral fusion did not change significantly, regardless of age or smoking history.
